# *catena*-Poly[[bis­(dimethyl­formamide-κ*O*)cadmium(II)]-di-μ_2_-dicyanamido-κ^4^ *N* ^1^:*N* ^5^]

**DOI:** 10.1107/S1600536809046364

**Published:** 2009-11-11

**Authors:** Jinfang Zhang

**Affiliations:** a Molecular Materials Research Center, Scientific Research Academy, School of Chemistry and Chemical Engineering, Jiangsu University, Zhenjiang 212013, People’s Republic of China

## Abstract

In the title compound, [Cd(C_2_N_3_)_2_(C_3_H_7_NO)_2_], the Cd^2+^ ion lies on an inversion center and adopts an octa­hedral coordination geometry, in which four N atoms from four different dicyanamide ligands lie in the equatorial plane and two dimethyl­formamide O atoms occupy the axial positions. The Cd atoms are connected by two dicyanamide ligands, resulting in a neutral chain propagating parallel to [010].

## Related literature

For architectures and topologies of metal-organic compounds, see: Eddaoudi *et al.* (2001 ▶); Zhang *et al.* (2008 ▶). For their potential applications, see: Banerjee *et al.* (2008 ▶); Zhang *et al.* (2007 ▶). For metal-organic compounds including dicyanamide ligands, see: Jensen *et al.* (1999 ▶); Zhang (2009 ▶).
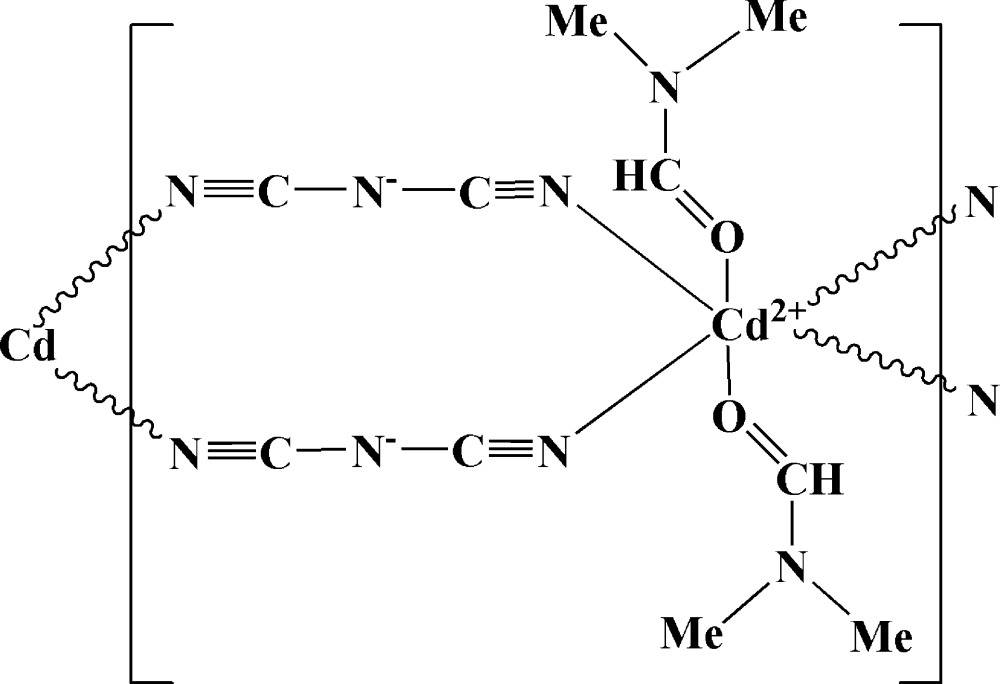


## Experimental

### 

#### Crystal data

[Cd(C_2_N_3_)_2_(C_3_H_7_NO)_2_]
*M* *_r_* = 390.70
Triclinic, 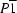

*a* = 6.5325 (13) Å
*b* = 7.6003 (15) Å
*c* = 8.6051 (17) Å
α = 104.28 (3)°
β = 106.90 (3)°
γ = 97.05 (3)°
*V* = 387.35 (17) Å^3^
*Z* = 1
Mo *K*α radiation
μ = 1.43 mm^−1^
*T* = 293 K
0.20 × 0.16 × 0.12 mm

#### Data collection

Rigaku Saturn724+ diffractometer
Absorption correction: multi-scan (*SADABS*; Sheldrick, 1996 ▶) *T*_min_ = 0.239, *T*_max_ = 0.480
3505 measured reflections
1410 independent reflections
1408 reflections with *I* > 2σ(*I*)
*R*_int_ = 0.025

#### Refinement

*R*[*F* ^2^ > 2σ(*F* ^2^)] = 0.022
*wR*(*F* ^2^) = 0.058
*S* = 1.10
1410 reflections
97 parameters
H-atom parameters constrained
Δρ_max_ = 0.44 e Å^−3^
Δρ_min_ = −0.36 e Å^−3^

### 

Data collection: *CrystalClear* (Rigaku, 2008 ▶); cell refinement: *CrystalClear*; data reduction: *CrystalClear*; program(s) used to solve structure: *SHELXS97* (Sheldrick, 2008 ▶); program(s) used to refine structure: *SHELXT07* (Sheldrick, 2008 ▶); molecular graphics: *SHELXTL* (Sheldrick, 2008 ▶); software used to prepare material for publication: *SHELXTL*.

## Supplementary Material

Crystal structure: contains datablocks I, global. DOI: 10.1107/S1600536809046364/pv2226sup1.cif

Structure factors: contains datablocks I. DOI: 10.1107/S1600536809046364/pv2226Isup2.hkl

Additional supplementary materials:  crystallographic information; 3D view; checkCIF report

## Figures and Tables

**Fig. 1.**
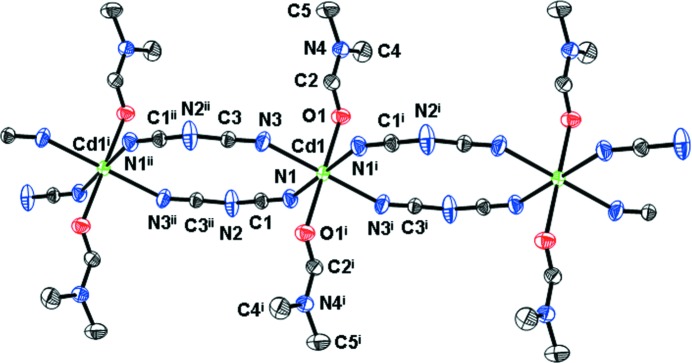
The molecular structure of the title compound, with atom labels and 30% probability displacement ellipsoids, all H atoms have been omitted (i -x + 1,-y + 1,-z; ii -x + 1,-y,-z).

## supplementary crystallographic information

### Comment

The designed syntheses of metal-organic compounds have attracted great attention in recent years because of not only their intriguing variety of architectures and topologies (Eddaoudi *et al.*, 2001; Zhang *et al.*, 2008) but also their potential applications (Banerjee *et al.*, 2008; Zhang *et al.*, 2007). Dicyanamide acting as flexible bridging ligands can construct metal-organic compounds with various structures (Jensen *et al.*, 1999; Zhang, 2009). The one-dimensional neutral compounds {Cd[N(CN)_2_]_2_(dmf)_2_}_n_ are constructed by this bridging ligands through diffusion reactions. In this paper, the crystal structure of the title compound, (I), is presented.

As illustrated in Fig. 1, Cd^2+^ which lies on an inversion center, adopts an octahedral coordination geometry, where four N atoms from four different dicyanamide ligands lies in equatorial plane and two O atoms from dmf occupy the axial positions. Every two neighboring Cd atoms connected by two dicyanamide ligands, gives rise to a one-dimensional neutral chain.

### Experimental

Cd(NO_3_)_2_.4 H_2_O (123.2 mg, 0.4 mmol) was added into 2 ml dmf with thorough stirring for 5 minutes. After filtration, the filtrate was carefully laid on the surface with the solution of NaN(CN)_2_ (89.1 mg, 1 mmol) in 1 ml dmf and 6 ml CH_3_CN. colorless block crystals were obtained after eight days. Yield: 199.3 mg in pure form, 51.0% based on Cd.

### Refinement

H atoms were positioned geometrically and refined with riding model, with *U*_iso_ = 1.5 and 1.2 *U*_eq_ for methyl and formyl H atoms, respectively. The C—H bonds are 0.96 Å in methyl and 0.93 Å in formyl.

### Crystal data

**Table:**

[Cd(C_2_N_3_)_2_(C_3_H_7_NO)_2_]	*Z* = 1
*M**_r_* = 390.70	*F*(000) = 194
Triclinic, *P*1	*D*_x_ = 1.675 Mg m^−^^3^
Hall symbol: -P 1	Mo *K*α radiation, λ = 0.71073 Å
*a* = 6.5325 (13) Å	Cell parameters from 1884 reflections
*b* = 7.6003 (15) Å	θ = 3.3–28.4°
*c* = 8.6051 (17) Å	µ = 1.43 mm^−^^1^
α = 104.28 (3)°	*T* = 293 K
β = 106.90 (3)°	Block, colorless
γ = 97.05 (3)°	0.2 × 0.16 × 0.12 mm
*V* = 387.35 (17) Å^3^	

### Data collection

**Table:**

Rigaku Saturn724+ diffractometer	1410 independent reflections
Radiation source: fine-focus sealed tube	1408 reflections with *I* > 2σ(*I*)
graphite	*R*_int_ = 0.025
dtprofit.ref scans	θ_max_ = 25.5°, θ_min_ = 3.3°
Absorption correction: multi-scan (*SADABS*; Sheldrick, 1996)	*h* = −7→7
*T*_min_ = 0.239, *T*_max_ = 0.480	*k* = −7→9
3505 measured reflections	*l* = −9→10

### Refinement

**Table:**

Refinement on *F*^2^	Primary atom site location: structure-invariant direct methods
Least-squares matrix: full	Secondary atom site location: difference Fourier map
*R*[*F*^2^ > 2σ(*F*^2^)] = 0.022	Hydrogen site location: inferred from neighbouring sites
*wR*(*F*^2^) = 0.058	H-atom parameters constrained
*S* = 1.10	*w* = 1/[σ^2^(*F*_o_^2^) + (0.039*P*)^2^ + 0.0503*P*] where *P* = (*F*_o_^2^ + 2*F*_c_^2^)/3
1410 reflections	(Δ/σ)_max_ < 0.001
97 parameters	Δρ_max_ = 0.44 e Å^−^^3^
0 restraints	Δρ_min_ = −0.36 e Å^−^^3^

### Special details

**Table:**

Geometry. All e.s.d.'s (except the e.s.d. in the dihedral angle between two l.s. planes) are estimated using the full covariance matrix. The cell e.s.d.'s are taken into account individually in the estimation of e.s.d.'s in distances, angles and torsion angles; correlations between e.s.d.'s in cell parameters are only used when they are defined by crystal symmetry. An approximate (isotropic) treatment of cell e.s.d.'s is used for estimating e.s.d.'s involving l.s. planes.
Refinement. Refinement of *F*^2^ against ALL reflections. The weighted *R*-factor *wR* and goodness of fit *S* are based on *F*^2^, conventional *R*-factors *R* are based on *F*, with *F* set to zero for negative *F*^2^. The threshold expression of *F*^2^ > σ(*F*^2^) is used only for calculating *R*-factors(gt) *etc*. and is not relevant to the choice of reflections for refinement. *R*-factors based on *F*^2^ are statistically about twice as large as those based on *F*, and *R*- factors based on ALL data will be even larger.

### Fractional atomic coordinates and isotropic or equivalent isotropic displacement parameters (Å^2^)

**Table:**

	*x*	*y*	*z*	*U*_iso_*/*U*_eq_	
Cd1	0.5000	0.5000	0.0000	0.04117 (11)	
O1	0.7512 (3)	0.6362 (3)	0.2708 (2)	0.0572 (5)	
N1	0.2979 (4)	0.3110 (3)	0.0987 (3)	0.0570 (6)	
N2	0.2301 (6)	0.0218 (4)	0.1653 (4)	0.0781 (9)	
N4	1.1012 (4)	0.7109 (3)	0.4447 (3)	0.0500 (5)	
N3	0.6991 (4)	0.2758 (3)	−0.0440 (3)	0.0598 (6)	
C1	0.2690 (4)	0.1705 (3)	0.1214 (3)	0.0449 (5)	
C5	1.3277 (5)	0.6980 (6)	0.4658 (4)	0.0755 (9)	
H5A	1.3381	0.6327	0.3586	0.113*	
H5B	1.3766	0.6322	0.5470	0.113*	
H5C	1.4177	0.8205	0.5059	0.113*	
C4	1.0524 (7)	0.8062 (5)	0.5930 (4)	0.0728 (9)	
H4A	0.8984	0.8039	0.5624	0.109*	
H4B	1.1323	0.9326	0.6357	0.109*	
H4C	1.0943	0.7454	0.6792	0.109*	
C2	0.9476 (4)	0.6352 (4)	0.2975 (3)	0.0468 (6)	
H2A	0.9885	0.5765	0.2063	0.056*	
C3	0.7261 (4)	0.1317 (3)	−0.0954 (3)	0.0427 (5)	

### Atomic displacement parameters (Å^2^)

**Table:**

	*U*^11^	*U*^22^	*U*^33^	*U*^12^	*U*^13^	*U*^23^
Cd1	0.04194 (16)	0.03209 (15)	0.04955 (17)	0.01065 (10)	0.01353 (11)	0.01291 (10)
O1	0.0480 (11)	0.0673 (13)	0.0488 (9)	0.0118 (9)	0.0137 (8)	0.0076 (9)
N1	0.0607 (14)	0.0420 (12)	0.0744 (15)	0.0081 (10)	0.0296 (12)	0.0209 (11)
N2	0.136 (3)	0.0421 (13)	0.0862 (18)	0.0263 (15)	0.077 (2)	0.0210 (13)
N4	0.0533 (12)	0.0530 (12)	0.0389 (10)	0.0105 (10)	0.0105 (9)	0.0115 (9)
N3	0.0660 (15)	0.0466 (13)	0.0771 (15)	0.0271 (11)	0.0296 (12)	0.0221 (11)
C1	0.0469 (13)	0.0378 (13)	0.0505 (13)	0.0082 (10)	0.0217 (10)	0.0080 (10)
C5	0.0518 (17)	0.100 (3)	0.0616 (18)	0.0162 (17)	0.0065 (14)	0.0165 (17)
C4	0.093 (2)	0.076 (2)	0.0426 (14)	0.0225 (18)	0.0204 (15)	0.0069 (14)
C2	0.0495 (14)	0.0473 (14)	0.0397 (12)	0.0070 (11)	0.0134 (10)	0.0096 (10)
C3	0.0457 (13)	0.0391 (13)	0.0461 (12)	0.0110 (10)	0.0167 (10)	0.0150 (10)

### Geometric parameters (Å, °)

**Table:**

Cd1—N3^i^	2.291 (2)	N4—C4	1.446 (4)
Cd1—N3	2.291 (2)	N4—C5	1.456 (4)
Cd1—N1	2.306 (2)	N3—C3	1.132 (3)
Cd1—N1^i^	2.306 (2)	C5—H5A	0.9600
Cd1—O1	2.316 (2)	C5—H5B	0.9600
Cd1—O1^i^	2.316 (2)	C5—H5C	0.9600
O1—C2	1.237 (3)	C4—H4A	0.9600
N1—C1	1.136 (3)	C4—H4B	0.9600
N2—C3^ii^	1.281 (4)	C4—H4C	0.9600
N2—C1	1.296 (4)	C2—H2A	0.9300
N4—C2	1.305 (3)	C3—N2^ii^	1.281 (4)
			
N3^i^—Cd1—N3	180.0	C4—N4—C5	117.7 (3)
N3^i^—Cd1—N1	91.27 (9)	C3—N3—Cd1	156.3 (2)
N3—Cd1—N1	88.73 (9)	N1—C1—N2	172.2 (3)
N3^i^—Cd1—N1^i^	88.73 (9)	N4—C5—H5A	109.5
N3—Cd1—N1^i^	91.27 (9)	N4—C5—H5B	109.5
N1—Cd1—N1^i^	180.00 (11)	H5A—C5—H5B	109.5
N3^i^—Cd1—O1	90.45 (9)	N4—C5—H5C	109.5
N3—Cd1—O1	89.55 (9)	H5A—C5—H5C	109.5
N1—Cd1—O1	91.26 (9)	H5B—C5—H5C	109.5
N1^i^—Cd1—O1	88.74 (9)	N4—C4—H4A	109.5
N3^i^—Cd1—O1^i^	89.55 (9)	N4—C4—H4B	109.5
N3—Cd1—O1^i^	90.45 (9)	H4A—C4—H4B	109.5
N1—Cd1—O1^i^	88.74 (9)	N4—C4—H4C	109.5
N1^i^—Cd1—O1^i^	91.26 (9)	H4A—C4—H4C	109.5
O1—Cd1—O1^i^	180.00 (11)	H4B—C4—H4C	109.5
C2—O1—Cd1	120.12 (16)	O1—C2—N4	124.5 (2)
C1—N1—Cd1	145.5 (2)	O1—C2—H2A	117.7
C3^ii^—N2—C1	122.3 (2)	N4—C2—H2A	117.7
C2—N4—C4	121.4 (3)	N3—C3—N2^ii^	172.2 (3)
C2—N4—C5	120.8 (2)		

Symmetry codes: (i) −*x*+1, −*y*+1, −*z*; (ii) −*x*+1, −*y*, −*z*.
